# Composition and diversity of butterflies (Lepidoptera, Papilionoidea) along an atmospheric pollution gradient in the Monterrey Metropolitan Area, Mexico

**DOI:** 10.3897/zookeys.1037.66001

**Published:** 2021-05-14

**Authors:** Edmar Meléndez-Jaramillo, César Martín Cantú-Ayala, Eduardo Javier Treviño-Garza, Uriel Jeshua Sánchez-Reyes, Bernal Herrera-Fernández

**Affiliations:** 1 Facultad de Ciencias Forestales, Universidad Autónoma de Nuevo León, Ap. Postal 41, Linares, Nuevo León, C.P. 67700, México Universidad Autónoma de Nuevo León Linares Mexico; 2 Tecnológico Nacional de México - Instituto Tecnológico de Cd. Victoria. Boulevard Emilio Portes Gil No.1301, C.P. 87010, Ciudad Victoria, Tamaulipas, México Instituto Tecnológico de Cd. Victoria Ciudad Victoria Mexico; 3 Fundación para el Desarrollo de la Cordillera Volcánica Central (Fundecor), Costa Rica e Instituto Internacional para la Conservación y Manejo de la Vida Silvestre (Icomvis), Universidad Nacional, Heredia, Costa Rica Universidad Nacional Heredia Costa Rica

**Keywords:** Atmospheric pollution, community patterns, indicator species, Papilionoidea, seasonality

## Abstract

This study compares the variation of richness, abundance and diversity of butterfly species along an atmospheric pollution gradient and during different seasons in the Monterrey Metropolitan Area, Mexico. Likewise, we analyse the influence of environmental variables on the abundance and richness of butterfly species and quantify the indicator species for each atmospheric pollution category. Based on spatial analysis of the main atmospheric pollutants and the vegetation cover conditions, four permanent sampling sites were delimited. The sampling was carried out monthly in each of the sites using aerial entomological nets and ten Van Someren-Rydon traps during May 2018 to April 2019. A total of 8,570 specimens belonging to six families and 209 species were collected. Both species richness and abundance were significantly different between all sites, except for the comparison between the moderate contamination site and the high contamination site; diversity decreased significantly with increasing levels of contamination. The seasonality effect was absent on species richness; however, for species abundance the differences between dry season and rainy season were significant in each site excepting the moderate contamination site. Regarding diversity, the seasonal effect showed different distribution patterns according to each order. Relative humidity, vegetation cover and three pollution variables were highly correlated with both abundance and species richness. From the total number of species found, only 47 had a significant indicator value. This study constitutes the first faunistic contribution of butterflies as indicators of the environmental quality of urban areas in Mexico, which will help in the development of strategies for the management, planning and conservation of urban biodiversity.

## Introduction

The dynamics of the demographic growth that cities are facing represents a serious threat to the environment, to the health and quality of life of its inhabitants (Vlahov and Galea 2002). The excessive exploitation of the natural resources, land use changes, industrial and urban concentrations and the large quantity of pollutants being emitted to the atmosphere, damage the environment in a process that seems irreversible (García et al. 2013). These effects not only harm living beings, but also generate phenomena that affect the ecosystem (López et al. 2001). This unregulated urbanisation process and ecosystems degradation occurs more rapidly in countries located in regions classified as developing economies, particularly in Latin America, where it is estimated that 75% of the population lives in cities (UN–HABITAT 2010).

In Mexico, atmospheric pollution has deteriorated air quality in various cities, including the Valle de México Metropolitan Zone, the Guadalajara Metropolitan Zone and the Monterrey Metropolitan Zone (MMZ) (García et al. 2012; Cerón et al. 2014; Mancilla et al. 2015; Menchaca et al. 2015). It should be noted that the main problem is the perception of society which often does not realise the severity of the problem, mainly because there is no clear awareness of pollutant emissions, their concentrations and the damage they cause to health, urban infrastructure, ecosystems and biodiversity (Lezama 2010). The State of Nuevo León, in the northeast of Mexico, has an unregulated urban growth. Its main city, the Monterrey Metropolitan Zone, presents serious environmental problems: geological and hydrological risks, water scarcity, green areas loss, air pollution, amongst many others (Cantú et al. 2013; Badillo et al. 2015; Orta et al. 2016; Sanchez-Castillo et al. 2016; Sisto et al. 2016; Sanchez-Castillo et al. 2017).

Studies on species diversity in urban ecosystems with air pollution problems are necessary to understand the effect of anthropogenic development on the integrity and livelihood of the ecosystem (Mukherjee et al. 2015). However, arthropods in such environments are poorly studied despite being crucial components and indicators of urban ecosystems and biodiversity (McIntyre 2000; Magle et al. 2012; Bonebrake and Cooper 2014). Butterflies, in general, are very sensitive to changes in temperature, humidity and solar radiation produced by disturbances in their habitat, for which the inventory of their communities, through measures of diversity and richness, represents a valid tool for evaluating the state of alteration between an urbanised area with pollution problems and a natural environment (Kremen 1993; Wagner et al. 2003; García et al. 2007; Settele et al. 2008; Li et al. 2009). In this way, these insects act as biological indicators, which can reflect the state of the biota in terms of biodiversity, its variation along gradients, endemism or the degree of human intervention, including air pollution (Fagua 2001; Moreno et al. 2007; Butchart et al. 2010; Defra 2016). Various studies demonstrate that butterfly species richness decreases as urbanisation increases (Blair and Launer 1997; Blair 1999; Hardy and Dennis 1999; Brown and Freitas 2002; Di Mauro et al. 2007; Konvička and Kadlec 2011; Bonebrake and Cooper 2014; Ramírez and MacGregor 2016), not only because the construction of buildings and roads replaces or reduces the area of natural and semi-natural habitats, but because the quality of residual habitats is affected by various forms of pollution (Corke 1998; Hardy and Dennis 1999; Mulder et al. 2005; Jones and Leather 2012; Philips et al. 2017).

Some other studies have yielded results that support the intermediate disturbance hypothesis, in which species diversity peaked in areas with an intermediate level of habitat alteration (Dial and Roughgarden 1998; Niell 2001; Giuliano et al. 2004; Koh and Sodhi 2004; Jones and Leather 2012). On the other hand, most insect studies investigating seasonality are based on relatively pristine ecosystems and few have examined the relationship between urban ecosystems seasonality and their butterfly assemblages. Only very few studies have explored the seasonality of urban butterflies, including Brown and Freitas (2002) in Sao Paulo, Brazil; Shapiro (2002) in California, USA; Koh and Sodhi (2004) in Singapore; Chowdhury et al. (2017) in Dhaka, Bangladesh; Gupta et al. (2019) in Delhi, India. The objectives presented here have the caveat that urban gradient studies are clearly a simplification of the complex patterns produced by urbanisation, such as pollution (Alberti et al. 2001; Hahs and McDonnell 2006; McKinney 2008). Therefore, the objectives of this study are: (1) Identify the butterfly species richness in the Monterrey Metropolitan Zone, Mexico; (2) Compare the variation in richness, abundance and diversity of butterfly species along an atmospheric pollution gradient and during the seasons of the year; (3) Analyse the influence of environmental variables (atmospheric pollutants, temperature, relative humidity, solar radiation and vegetation cover) on the abundance and richness of butterfly species; and (4) quantify the indicator value of the species per atmospheric pollution category.

## Methods

### Study area

**Figure 1.**
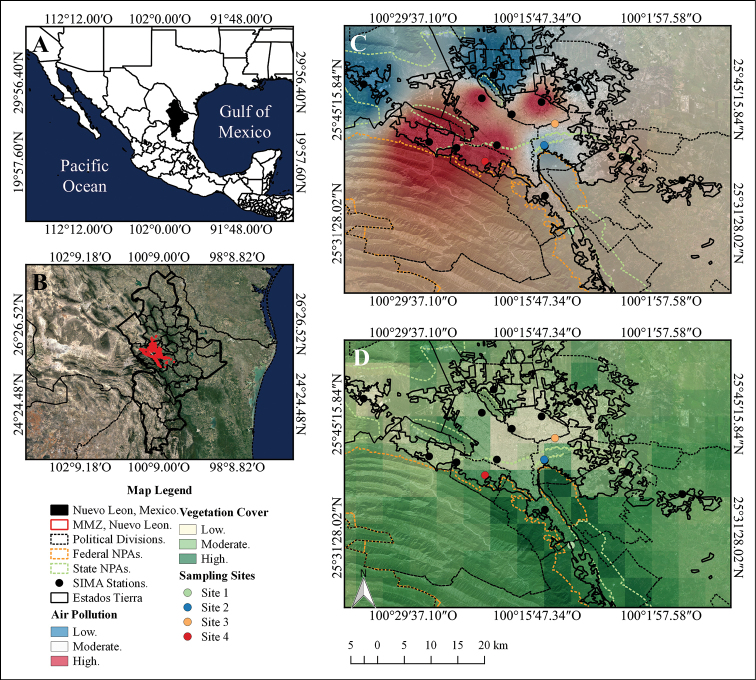
Study area and location of sampling sites **A** location of Nuevo Leon in Mexico **B** location of MMZ inside of Nuevo Leon **C** location of the sampling sites according to the level of atmospheric pollutant **D** location of the sampling sites according to the levels of vegetation cover.

The Monterrey Metropolitan Zone (**MMZ**) is the largest urban area in northeast Mexico and the third largest urban centre in the country, extending from 25°15' to 26°30' of north latitude and from 99°40' to 101°10' of west longitude (Figure 1A, B). The area is limited by the coastal plain of the Gulf of Mexico and the Sierra Madre Oriental mountain range. The MMZ urban sprawl integrates the Municipality of Monterrey in the central portion, the Municipalities of Guadalupe, San Nicolas de los Garza and San Pedro Garza Garcia in the pericentral portion, Apodaca, Escobedo and Santa Catarina Municipalities in the periphery and El Carmen, Garcia, Santiago, Juarez, Cadereyta and Salinas Victoria in the surrounding area (Alanís 2005; González et al. 2011; Mancilla et al. 2015; Ybáñez and Barboza 2017). The MMZ has a vehicle fleet of 1.7 million (INEGI 2010) and 4.1 million of inhabitants (INEGI 2011), which is probably higher nowadays. Likewise, there is a variety of industrial complexes that include the production of glass, steel, cement and paper, amongst others (Menchaca et al. 2015). The centre of the city has an average altitude of 540 m a.s.l, its steppe climate is dry and warm with temperatures above 35 °C during the summer and below 8 °C during the winter (Alanís 2005; González et al. 2011; Menchaca et al. 2015).

### Delimitation of the pollution and vegetation cover gradients

Since November 1992, the MMZ operates a network of air quality monitoring stations known as the Integral Environmental Monitoring System (SIMA). The SIMA network currently consists of 13 registration stations, located following the criteria of meteorological, epidemiological, land use and population density studies. The concentrations registered in the monitoring stations are: PM_10_ (particulate matter of less than 10 µm), PM_2.5_ (particulate matter of less than 2.5 µm), carbon monoxide (CO), ozone (O_3_), nitrogen dioxide (NO_2_), nitrogen oxides (NO_x_) and sulphur dioxide (SO_2_). In addition, some meteorological variables are reported, such as barometric pressure (Bp), rainfall (R), relative humidity (Rh), solar radiation (Sr), temperature (T) and the direction (Wd) and magnitude of wind (Ws) (Arreola and González 1999; González et al. 2011; Mancilla et al. 2015). The data recorded by SIMA stations for air quality and meteorological variables during the period from 2008 to 2017 were obtained from the website of the National System of Air Quality Information (SINAICA). Descriptive measures for each of the months and each registered year were obtained in the Statistica 13.3 software (TIBCO Software Inc. 2017).

To identify the main air quality descriptor pollutants in the MMZ during the period 2008–2017, a Principal Component Analysis (PCA) was carried out. Subsequently, to differentiate the changes in the spatial distribution of the air quality indicator pollutants in the MMZ, maps were prepared using the annual average information per monitoring station. The creation of maps was carried out using Inverse Distance Weighting Interpolation (IDW), with a value of 2 as Coefficient of Distance and the pixel size of the output raster re-defined to 10 metres. As reference of the extension for each interpolation, the minimum and maximum distances were taken from the vector sections corresponding to the urban areas that form the MMZ; such vectors were obtained from the National Land Use and Vegetation Series 6 layer (INEGI 2016). The procedures described above were performed using the QGis 3.2 software (QGIS Development Team 2018). As a result, three categories of air pollution were generated: low (0.19 to 26.51 µg/m^3^ of NO_x_; 3.22 to 10.56 µg/m^3^ of PM_2.5_), moderate (26.51 to 52.83 µg/m^3^ of NO_x_; 10.56 to 17.92 µg/m^3^ of PM_2.5_) and elevated (52.83 to 79.19 µg/m^3^ of NO_x_; 17.92 to 25.3 µg/m^3^ of PM_2.5_) (Figure 1C).

Percentage of vegetation cover was determined through an analysis of MODIS images for the period 2008–2017, obtained from GIOVANNI online server. Consequently, three categories of vegetation cover were designated: low (23 to 40%), moderate (40 to 57%) and high (57 to 74%) (Figure 1D).

### Selection of sampling sites

**Figure 2.**
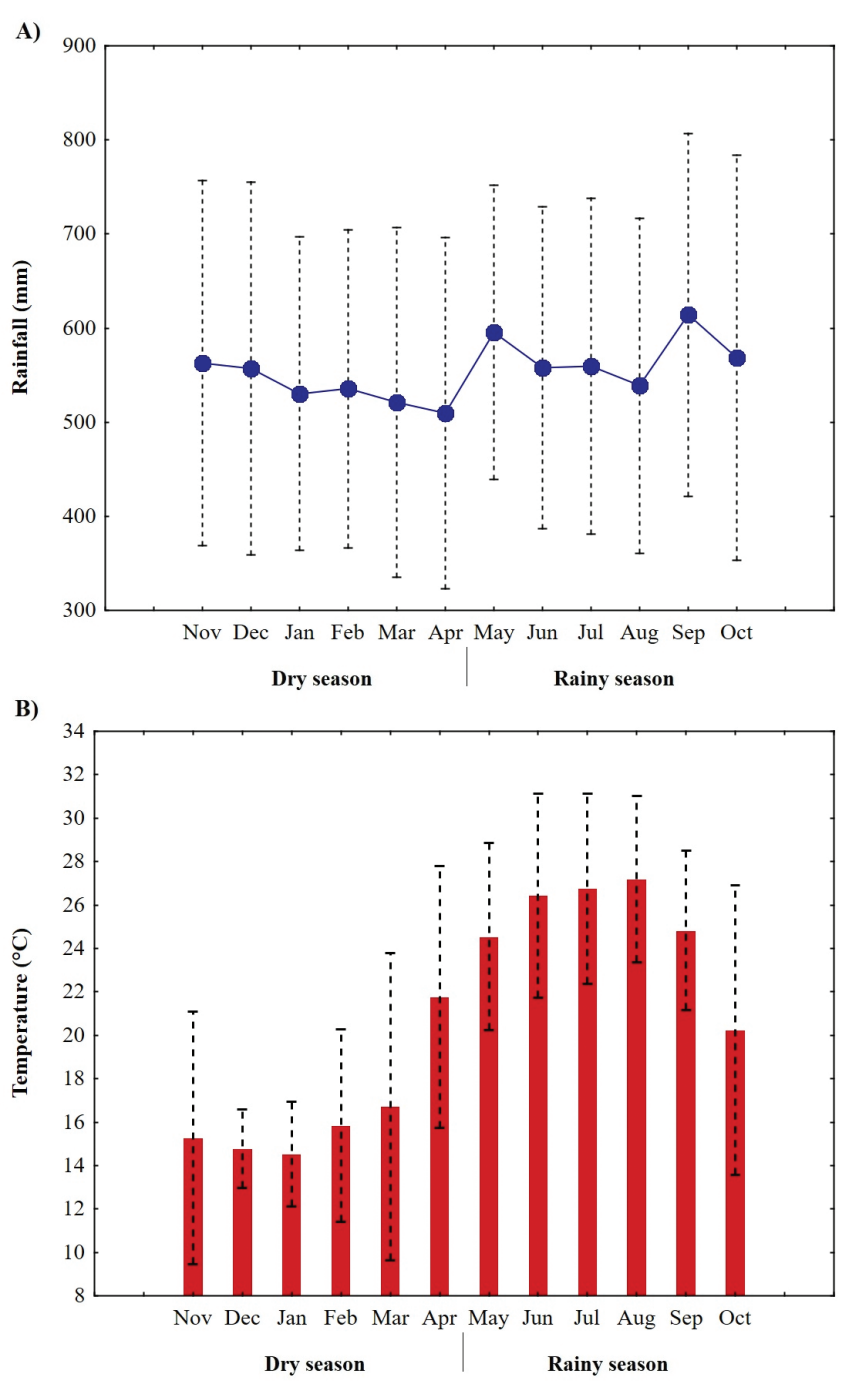
Average monthly variation of temperature and accumulated rain in the MMZ.

**Figure 3.**
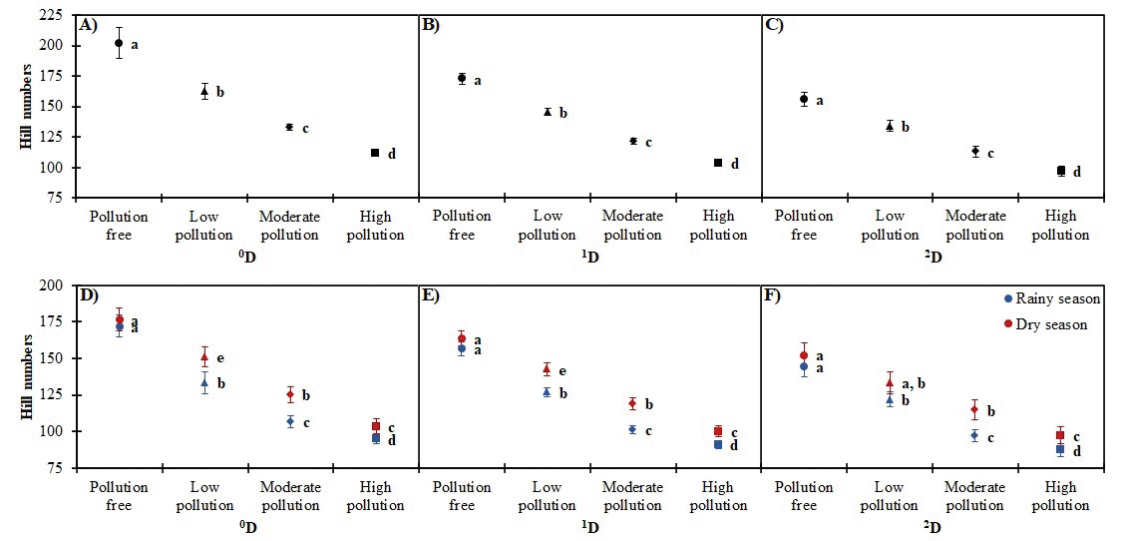
Alpha diversity profiles (^0^D, ^1^D and ^2^D) of butterflies along the pollution gradient and by season (dry and rainy) in the MMZ. The error bars represent 95% confidence intervals.

Four permanent sampling sites were delimited, based on the spatial overlapping of four geographic elements: (1) the IDW analysis of the main atmospheric pollutants (Figure 1C), (2) the vegetation cover (Figure 1D), (3) images obtained from Google Earth Pro software and (4) a mesh with a grid size of 150 × 150 metres. The procedures of superimposition and selection were performed in QGis 3.2 software. Site 1 is found in the Municipality of Santiago, an atmospheric pollution-free area with secondary vegetation of submontane scrub (25°30'41.184"N, 100°11'53.159"W). Site 2 is located in the central zone of the Municipality of Guadalupe with low values of atmospheric pollution and secondary vegetation of submontane scrub (25°40'4.944"N, 100°14'45.564"W). Site 3 is located in the northern zone of the Municipality of Guadalupe with moderate atmospheric pollution and secondary vegetation of submontane scrub (25°42'44.017"N, 100°13'58.825"W). Site 4 is in the Municipality of San Pedro Garza Garcia with high atmospheric pollution and anthropogenic vegetation of submontane scrub (25°38'11.112"N, 100°21'30.815"W) (Table 1, Figure 1).

**Table 1.** Descriptive synthesis of the sampling sites.

Site	Vegetation	Frequent species	General description
1	Secondary submontane scrub	*Ehretia anacua*, *Ebenopsis ebano*, *Havardia pallens*, *Prosopis glyulosa*, *Celtis laevigata*, *Sideroxylon celastrinum* and *Eragrostis barrelieri*.	Vacant site located in the Municipality of Santiago, with elevation of 530 m a.s.l. The site is outside the limits of registration of atmospheric pollution and with vegetation cover of 71.06%.
2	Secondary submontane scrub	*Ehretia anacua*, *Ebenopsis ebano*, *Prosopis glyulosa*, *Fraxinus americana*, *Celtis laevigata*, *Leucaena leucocephala* and *Euphorbia hirta*.	Site inside La Pastora Park Zoo in the Municipality of Guadalupe. Elevation of 492 m a.s.l, as well as low levels of atmospheric pollution and a vegetation cover of 53.47%.
3	Secondary submontane scrub	*Ebenopsis ebano*, *Leucaena leucocephala*, *Fraxinus americana*, *Cordia boissieri*, *Parkinsonia aculeata*, *Caesalpinia mexicana* and *Eragrostis barrelieri*.	Vacant site in the northern limit of the Municipality of Guadalupe, at an elevation of 486 m a.s.l. It presents moderate levels of atmospheric pollution, and a vegetation cover of 46.3%.
4	Anthropogenic submontane scrub	*Fraxinus americana*, *Ligustrum lucidum*, *Populus tremuloides* and *Phyla nodiflora*.	Abandoned square in the Municipality of San Pedro Garza García. Site with an elevation of 663 m a.s.l., high levels of atmospheric pollution and a vegetation cover of 58.03%.

### Sampling and processing of specimens

Monthly samplings were carried out for each of the sites, during the period from May 2018 to April 2019, resulting in a total of six samplings per season: dry season (November, December, January, February, March and April) and rainy season (May, June, July, August, September and October). The seasons were defined based on historical data of monthly total values of temperature and rain (average from 2008 to 2017), which were obtained from the SIMA stations located within the study area (Figure 2). Therefore, a total of 48 samplings were considered (six samplings for two seasons and four sites).

The sampling of individuals was carried out using an entomological aerial net. In each of the sites, tours were made inside a pre-established quadrant of 150 m × 150 m, following the techniques recommended by Villarreal et al. (2006). The sampling time at each site was nine hours in the period from 08:00 h to 17:00 h. Furthermore, along with the use of aerial nets, Van Someren-Rydon traps were used (Rydon 1964). Ten traps were placed, five at one end of the quadrant and five at the opposite end, at a distance of 30 m from each other and between 1 and 2.5 m from the ground, with an exposure time of nine hours (08:00 h to 17:00 h). The bait used for the traps consisted of a fermented mixture of seasonal fruits: banana (*Musa paradisiaca*), pineapple (*Ananas comosus*), mango (*Mangifera indica*) and guava (*Psidium guajava*).

The collected specimens were mounted according to the described procedure of Andrade et al. (2013). For taxonomic identification of specimens, the works of Scott (1986), Llorente et al. (1997), Luis et al. (2003), Garwood and Lehman (2005), Vargas et al. (2008), Luis et al. (2010) and Glassberg (2018) were consulted. The interactive list and the phylogenetic ordering of Warren et al. (2012) were taken as reference. All the specimens were labelled and deposited in the entomological collection of the Conservation Department of the Forestry Sciences Faculty at the Autonomous University of Nuevo León, Linares, Nuevo León, Mexico.

### Data analysis

The observed species richness was measured as the total number of species in the study area, as well as at each of the sites. The constancy index was determined and the species were classified as: constant (species found more than 50% of the time during sampling), accessory (species present between 25 to 50%) and accidental (species in less than 25%) (Sackis and Morais 2008). Significant differences in the number of species between sites were determined using a generalised linear model (GLM) (they were modelled as Poisson-distributed variables with a log link) and one-way ANOVA, with Statistica software 13.3. The sampling efficiency was calculated for the entire study area and for each study site using the interpolation and extrapolation methodology proposed by Chao and Jost (2012), available in the package iNEXT (Hsieh et al. 2016).

Five species categories were considered according to the total registered abundance: rare (species with one individual), scarce (from 2 to 5), frequent (from 6 to 21), common (from 22 to 81) and abundant (with 82 or more individuals) (Luna et al. 2010). Differences in the abundance of butterfly communities at the sites were calculated with a GLM and one-way ANOVA. For the analysis of alpha diversity, we adopted the analytical method of Chao and Jost (2015) to obtain diversity profiles in which diversity is evaluated in terms of “effective numbers of species” (qD), an approach that is equivalent to Hill's numbers (Hill 1973). The analysis was made for all the study area and for each study site using the 3.5.3 version of R (R Development Core Team 2019), with the package SpadeR (Chao et al. 2016). To examine the differences in species composition amongst the four sites, we performed a non-metric multidimensional scaling analysis (NMDS), based on the similarity matrix using the Bray-Curtis Index. A one-way PERMANOVA was also performed to test for differences in species composition between sites. Both analyses were performed using R 3.5.3 with the package Vegan (Oksanen et al. 2019).

The seasonal effect was measured separately, comparing the species richness, abundance and diversity observed per study site during the rainy season (May to October 2018) and dry season (November of 2018 to April 2019). The indexes and statistical tests mentioned above were used for such comparisons: GLM and nested ANOVA tests for differences in species richness and abundance, estimation of species richness and alpha diversity index, which were performed in Statistica 13.3 and R 3.5.3. In addition, a two-way PERMANOVA and NMDS analyses were carried out, to include the seasonal effect in the species composition, with the aim of grouping sites and seasons. These analyses were performed in Statistica 13.3 and R 3.5.3. A Canonical Correlation Test was applied between the community parameters (number of species and abundance) and the different environmental variables: monthly averages of the main variables of atmospheric pollution (with the highest loading values in the PCA previously obtained) (NO_2_, NO_x_, CO and PM_2.5_), climatological variables (temperature, relative humidity and solar radiation) and vegetation cover variables extracted from the SIMA stations and from MODIS images nearest to the sampling sites, using Statistica 13.3.

Finally, to calculate the association value of each butterfly species with the habitat type, the Indicator Value Index, or IndVal, was used (Dufrêne and Legendre 1997). This index is based on the degree of specificity (exclusivity of the species to a particular site, based on its abundance) and the degree of fidelity (frequency of occurrence within the same habitat) (Tejeda et al. 2008), expressed in a percentage value. The analysis was performed with the labsdv package on platform R 3.5.3, using 1,000 random permutations to define the level of significance. The indicator species with an index equal to or greater than 75% were classified as “characteristics”, which are defined by their high specificity for a given habitat; species with a value less than 75%, but equal to or greater than 50% are considered as “detectors”, which show different degrees of preference for diverse habitats (McGeoch et al. 2002).

## Results

### Variation of butterflies per pollution category

A total of 8,570 Papilionoidea specimens were collected, distributed in six families, 19 subfamilies, 31 tribes, 138 genera and 209 species. From this total, only 26 species (499 individuals) were registered exclusively with Van Someren-Rydon baited traps, while the remaining 183 species (8,071 individuals) were collected with entomological nets (Appendix 1). Nymphalidae was the most abundant family with 3,008 individuals, which represents 35.1% of total abundance in the study area. A lower abundance was recorded in Hesperiidae (23.2%), Pieridae (19.5%), Lycaenidae (11.9%), Papilionidae (6.6%) and Riodinidae (3.6%). The highest species richness was found in the Hesperiidae family with 32.5% of the total obtained species, followed by Nymphalidae (31.1%), Lycaenidae (14.4%), Pieridae (11%), Papilionidae (6.7%) and Riodinidae (4.3%). Twenty-two species were categorised as abundant (with more than 82 individuals) and represented 25.9% of the total abundance. *Kricogonia lyside* (Godart, 1819) (142 individuals), *Anaea aidea* (Guérin-Méneville, 1844) (122), *Phoebis sennae marcellina* (Cramer, 1777) (120), *Pyrisitia proterpia* (Fabricius, 1775) (117) and *Libytheana carinenta larvata* (Strecker, 1878) (113), amongst others, showed the highest abundance. One hundred and three species were considered as common, being 65.2% of the total number of individuals recorded. Fifty-four species were considered as frequent, occupying 8.2% of the total abundance. Seventeen were scarce (0.7% of total abundance) and three were rare (0.04%) (Appendix 1). On the other hand, 93 species (44.5%) were categorised as constant, 47 (22.5%) were accessory species and 69 (33%) were accidental. *Kricogonia lyside* and *Phoebis sennae marcellina* were the most frequent species (87.5%) during all the samplings.

The sample coverage estimator indicated that our inventory for the MMZ is 99.7% complete. In Figure 3, we plotted the proposed diversity with the method of Chao and Jost (2015) and the confidence intervals for q = 0, 1 and 2 for each pollution category and station.

**Figure 4.**
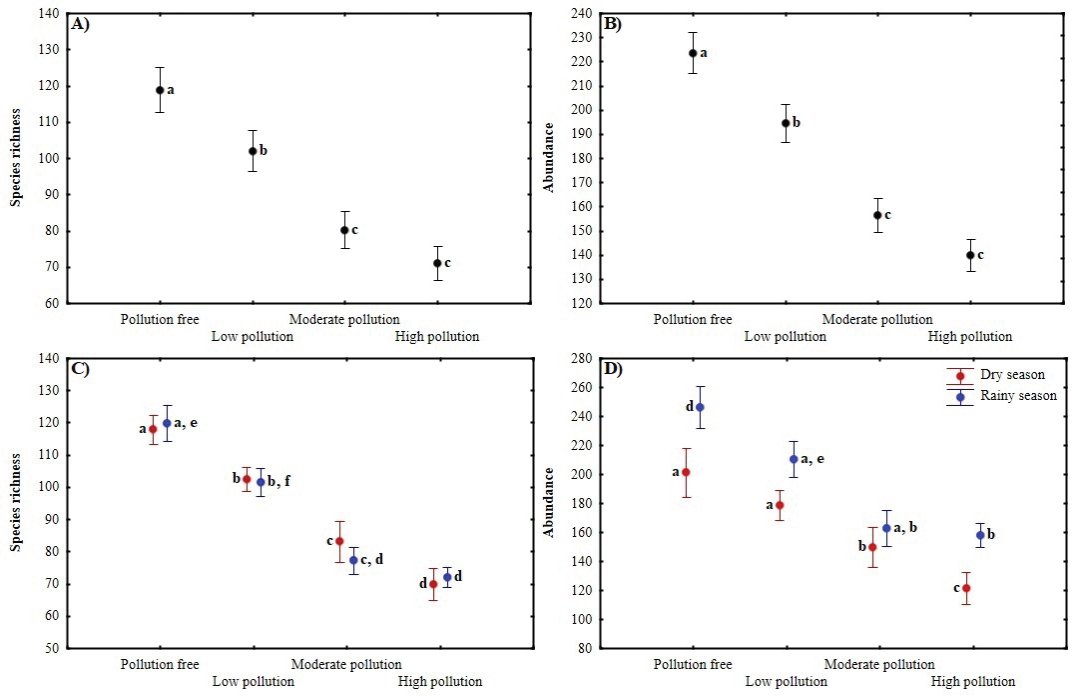
Effect of pollution and seasonality on species richness and abundance of butterflies (mean ± standard errors) of the MMZ. Different letters represent significant differences between categories (*p* < 0.05).

Both species richness and abundance were significantly different (*p* < 0.05) between all sites, except for the comparison between Site 3 (moderate pollution) and Site 4 (high pollution) (Table 2, Figure 4A, B). Both parameters (species richness and abundance) decreased with increasing levels of pollution. In Site 1 (pollution free) 2,683 individuals and 199 species were registered, representing a sampling coverage of 99.7%. In Site 2 (low pollution), the values were reduced to 2,334 individuals and 162 species (coverage of 99.9%). For Site 3 (moderate pollution), 1,876 individuals and 133 species were registered (coverage of 99.9%), while for Site 4 (high pollution), 1,677 individuals and 112 species (coverage of 100%).

**Table 2.** Summary of ANOVA (GLM) results for butterfly abundance and species richness by pollution categories in the MMZ.

Pollution categories	Estimate	95%Lower	95%Upper	p-Value
**Species richness**				
Intercept				
Pollution free	0.266	0.218	0.313	< 0.001
Low pollution	0.112	0.063	0.162	< 0.001
Moderate pollution	-0.128	-0.182	-0.074	< 0.001
**Species abundance**				
Intercept	5.168	5.146	5.190	< 0.001
Pollution free	0.242	0.207	0.276	< 0.001
Low pollution	0.102	0.067	0.138	< 0.001
Moderate pollution	-0.116	-0.155	-0.077	< 0.001

**Figure 5.**
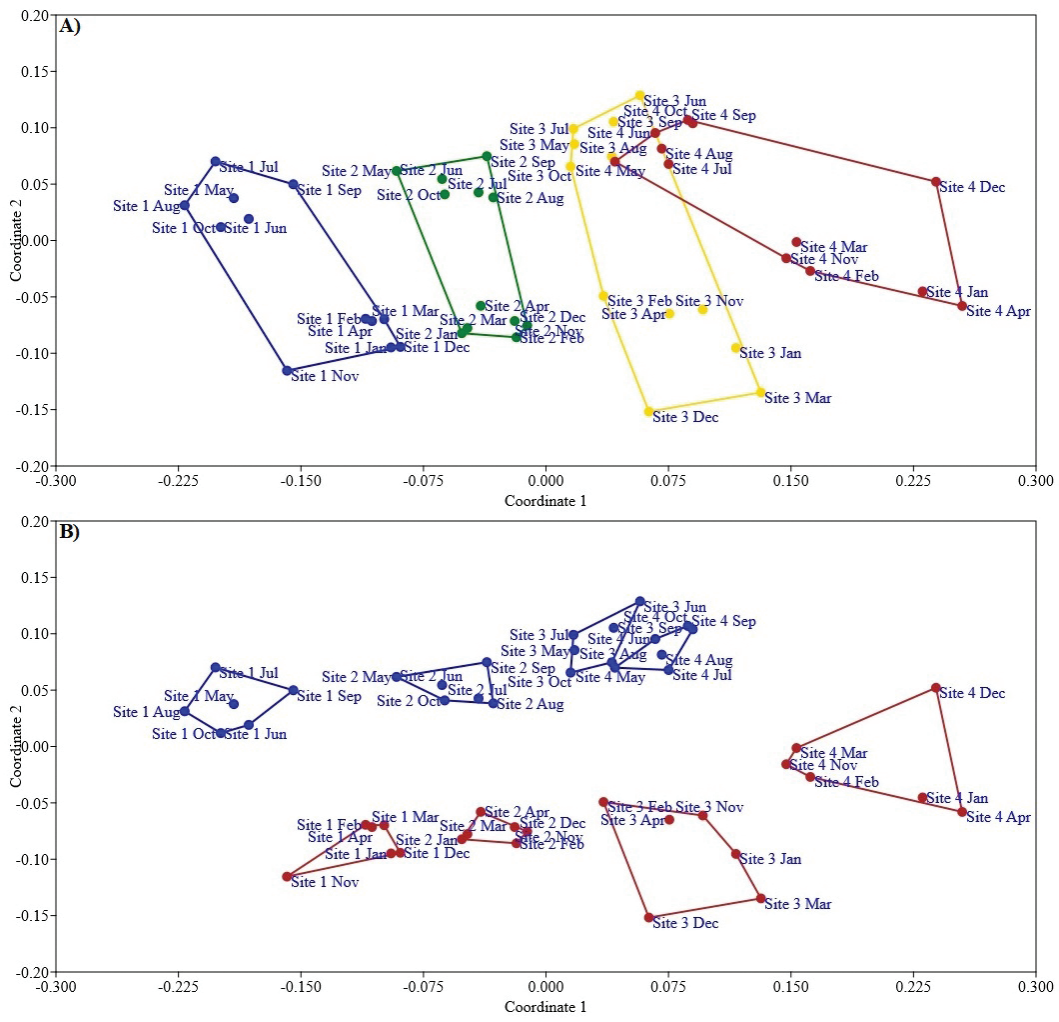
Non-metric multidimensional scaling ordination (NMDS) of the butterfly communities for contamination categories and seasons of the year of the MMZ**A** pollution free (blue colour), low pollution (green colour), moderate pollution (yellow colour) and high pollution (red colour) **B** rainy season (blue colour) and dry season (red colour).

For ^0^D, ^1^D and ^2^D, Site 1 (pollution free) had the highest diversity. All comparisons between sites were significantly different (with 95% confidence intervals) (Figure 3A, B and C). The one-way PERMANOVA test detected significant differences in species composition amongst Sites 1, 2 and 3 (free, low and moderate contamination) (SS_total_ = 6.63; SS_within-group_ = 5.05; F = 4.575, *p* < 0.001). Butterflies collected during each month for Sites 1, 2 and 3 (free, low and moderate contamination) formed separate groups in the NMDS diagram (Stress = 0.23) (Figure 5A).

### Effect of seasonality on butterfly changes per pollution category

In the MMZ, the highest abundance was registered during the rainy season, while the highest species richness was shown during the dry season. The highest completeness of the inventory was recorded during the rainy season (Table 2).

The differences in the abundance of the dry and rainy season were significant (*p* < 0.05) in each site with the exception of Site 3 (moderate contamination) (Table 3, Figure 4D). Regarding diversity, the seasonal effect was absent in Site 1 (pollution free) for ^0^D and ^1^D, while for ^2^D, Site 1 (pollution free), 2 (low contamination) and 4 (high contamination) did not show significant differences between seasons (Figure 3D, F).

**Table 3.** Summary of the nested ANOVA (GLM) for the species richness and abundance of butterflies by categories of contamination and seasons of the year in the MMZ.

Pollution categories	Estimate	95% Lower	95% Upper	p-Value
**Species richness**				
Intercept	4.277	4.182	4.371	< 0.001
Dry season (Pollution free)	-0.017	-0.121	0.087	> 0.05
Rainy season (Pollution free)	0.000			
Dry season (Low pollution)	0.008	-0.104	0.120	> 0.05
Rainy season (Low pollution)	0.000			
Dry season (Moderate pollution)	0.075	-0.052	0.201	> 0.05
Rainy season (Moderate pollution)	0.000			
Dry season (High pollution)	-0.031	-0.165	0.104	> 0.05
Rainy season (High pollution)	0.000			
**Species abundance**				
Intercept	5.063	4.999	5.126	< 0.001
Dry season (Pollution free)	-0.201	-0.277	-0.125	< 0.001
Rainy season (Pollution free)	0.000			
Dry season (Low pollution)	-0.165	-0.246	-0.083	< 0.001
Rainy season (Low pollution)	0.000			
Dry season (Moderate pollution)	-0.083	-0.174	0.007	> 0.05
Rainy season (Moderate pollution)	0.000			
Dry season (High pollution)	-0.263	-0.359	-0.166	< 0.001
Rainy season (High pollution)	0.000			

Two-way PERMANOVA allowed us to identify a significant effect of season (F = 7.702, df = 1, *p* < 0.001) and pollution (site) (F = 5.682, df = 3, *p* < 0.001) on species composition, as well as an interaction effect between the two factors (F = 2.315, df = 3, *p* < 0.001). Butterflies collected each month in each of the study sites formed separated groups by seasons in the NMDS ordination diagram (Stress = 0.23) (Figure 5B).

The variables NO_2_, NO_x_, PM_2.5_, relative humidity and vegetation cover were highly correlated, both with abundance and species richness. The individual interaction of NO_2_, NO_x_, PM_2.5_ and relative humidity with abundance and species richness resulted negative, while the interaction of vegetation cover with both ecological parameters resulted positive (Table 4).

**Table 4.** Correlation analysis between environmental variables with the abundance and richness of butterfly species in the MMZ. The marked correlations (*) are significant (*p <* 0.05).

	Abundance	Species richness
CO (ppm)	0.046	0.137
NO_2_ (ppm)	-0.725 *	-0.590 *
NO_x_ (ppm)	-0.595 *	-0.418 *
PM_2.5_ (µg/m³)	-0.652 *	-0.580 *
Temperature (°C)	-0.003	0.047
Relative humidity (%)	-0.487 *	-0.603 *
Solar radiation (Klux)	-0.007	0.027
Vegetation cover (%)	0.492 *	0.481 *

### Indicator value of butterflies in a gradient of pollution

From the 209 species found in the study area, only 47 had a significant indicator value (p < 0.05). The highest proportion included detector species, with an IndVal between 50 and 75% (30 species). The remaining 17 were characteristic, with values greater than 75% (Table 5). *Anteros carausius* Westwood, 1851 was considered the only indicator (detector) species at Site 4 (high pollution). In Site 2 (low pollution), the species *Polygonia interrogationis* (Fabricius, 1798), *Lasaia agesilas callaina* Clench, 1972; *Cyanophrys miserabilis* (Clench, 1946), *Panoquina lucas* (Fabricius, 1793), *Strymon yojoa* (Reakirt, 1867) and *Heraclides thoas autocles* Rothschild & Jordan, 1906 were considered as detector indicator species. Likewise, 23 species were considered as detector indicators of Site 1 (free pollution), amongst which, *Quinta cannae* (Herrich-Schäffer, 1869) and *Memphis pithyusa pithyusa* (R. Felder, 1869) have the highest values of IndVal. In Site 1, 17 species were considered as characteristic indicator species, those being *Pyrisitia dina westwoodii* (Boisduval, 1836), *Heliconius erato petiverana* (E. Doubleday, 1847) and *Timochares ruptifasciata* (Plötz, 1884) with the highest IndVal (Table 5).

**Table 5.** Butterfly species with a significant indicator value in the pollution gradient of the MMZ. Index values are expressed as a percentage. Legend: C = characteristic; D = detector; *p* = probability. The marked species (*) were collected with Van Someren-Rydon traps.

Taxon	Site 1	Site 2	Site 3	Site 4	*p*	Indicator category
*Anteros carausius carausius* Westwood, 1851	0.0	0.0	18.2	53.0	0.001	D
*Polygonia interrogationis (Fabricius, 1798)	2.8	69.4	0.0	0.0	0.000	D
*Lasaia agesilas callaina* Clench, 1972	20.8	63.9	0.0	0.0	0.004	D
*Cyanophrys miserabilis* (Clench, 1946)	18.2	60.6	0.0	0.0	0.017	D
*Panoquina lucas* (Fabricius, 1793)	9.5	59.5	0.0	0.0	0.001	D
*Strymon yojoa* (Reakirt, 1867)	13.1	51.8	0.0	0.0	0.018	D
*Heraclides thoas autocles* Rothschild & Jordan, 1906	31.7	51.6	0.0	0.0	0.001	D
*Quinta cannae* (Herrich-Schäffer, 1869)	75.0	8.3	0.0	0.0	0.000	D
*Memphis pithyusa pithyusa (R. Felder, 1869)	68.5	0.0	0.0	0.0	0.001	D
*Protographium epidaus epidaus* (Doubleday, 1846)	66.7	0.0	0.0	0.0	0.001	D
*Heraclides anchisiades idaeus* Fabricius, 1793	66.7	0.0	0.0	0.0	0.013	D
*Atlides halesus corcorani* Clench, 1942	66.7	0.0	0.0	0.0	0.001	D
*Michaelus hecate* (Godman y Salvin, 1887)	66.7	0.0	0.0	0.0	0.000	D
*Temenis laothoe (Cramer, 1777)	66.7	0.0	0.0	0.0	0.001	D
*Thorybes pylades albosuffusa* H. Freeman, 1943	66.7	0.0	0.0	0.0	0.001	D
*Autochton cellus* (Boisduval & Le Conte, 1837)	66.7	0.0	0.0	0.0	0.018	D
*Calephelis rawsoni* McAlpine, 1939	64.8	11.1	0.0	0.0	0.020	D
*Asterocampa idyja argus (H. Bates, 1864)	64.3	21.4	0.0	0.0	0.001	D
*Strymon bazochii bazochii* (Godart, 1824)	61.1	0.0	0.0	0.0	0.001	D
*Limenitis arthemis astyanax (Fabricius, 1775)	59.5	0.0	0.0	0.0	0.021	D
*Eurema daira eugenia* (Wallengren, 1860)	56.9	35.9	0.0	0.0	0.002	D
*Anteos clorinde* (Godart, 1824)	56.8	23.8	0.0	0.0	0.002	D
*Polyctor enops* (Godman & Salvin, 1894)	56.7	0.0	0.0	0.0	0.006	D
*Anthanassa tulcis* (H. Bates, 1864)	55.2	37.4	0.0	0.0	0.000	D
*Rekoa zebina* (Hewitson, 1869)	54.8	19.0	0.0	0.0	0.011	D
*Eurema boisduvaliana* (C. Felder & R. Felder, 1865)	51.0	10.3	25.6	0.0	0.004	D
*Megisto rubricata rubricata (W. H. Edwards, 1871)	50.0	0.0	0.0	0.0	0.009	D
*Carrhenes canescens canescens* (R. Felder, 1869)	50.0	0.0	0.0	0.0	0.008	D
*Wallengrenia otho otho* (J. E. Smith, 1797)	50.0	0.0	0.0	0.0	0.009	D
*Anatrytone mazai* (H. Freeman, 1969)	50.0	0.0	0.0	0.0	0.009	D
*Pyrisitia dina westwoodii* (Boisduval, 1836)	100.0	0.0	0.0	0.0	0.000	C
*Heliconius erato petiverana* (E. Doubleday, 1847)	100.0	0.0	0.0	0.0	0.000	C
*Timochares ruptifasciata* (Plötz, 1884)	100.0	0.0	0.0	0.0	0.000	C
*Eumaeus childrenae* (G. Gray, 1832)	90.7	0.0	0.0	0.0	0.000	C
*Dymasia dymas dymas* (W. H. Edwards, 1877)	90.7	0.0	0.0	0.0	0.002	C
*Leptophobia aripa elodia* (Boisduval, 1836)	90.4	0.0	0.0	0.0	0.001	C
*Cyanophrys herodotus* (Fabricius, 1793)	83.3	0.0	0.0	0.0	0.000	C
*Anthanassa ardys* (Hewitson, 1864)	83.3	0.0	0.0	0.0	0.004	C
*Sostrata nordica* Evans, 1953	83.3	0.0	0.0	0.0	0.000	C
*Parides erithalion polyzelus* (C. Felder & R. Felder, 1865)	83.3	0.0	0.0	0.0	0.000	C
*Allosmaitia strophius* (Godart, 1824)	83.3	0.0	0.0	0.0	0.003	C
*Strymon bebrycia* (Hewitson, 1868)	83.3	0.0	0.0	0.0	0.000	C
*Tmolus echion echiolus* (Draudt, 1920)	83.3	0.0	0.0	0.0	0.000	C
*Heliopetes macaira macaira* (Reakirt, 1867)	83.3	0.0	0.0	0.0	0.000	C
*Cymaenes trebius* (Mabille, 1891)	83.3	0.0	0.0	0.0	0.000	C
*Staphylus azteca* (Scudder, 1872)	83.3	0.0	0.0	0.0	0.003	C
*Pterourus palamedes leontis* Rothschild & Jordan, 1906	76.5	0.0	0.0	0.0	0.001	C

## Discussion

This study constitutes the first faunistic contribution of butterflies as indicators of the environmental quality of an urban area in Mexico and the first inventory of butterflies systematically carried out in the State of Nuevo León. The 209 registered species in the MMZ constitute 78.6% of the described richness so far in Nuevo Leon, according to Luz and Madero (2011) in collaboration with the North American Butterfly Association (NABA) and 10.2% to what was recorded for Mexico (Warren 2000; Llorente et al. 2006). However, it should be noted that urban gradient studies are clearly a simplification of the complex patterns produced by urbanisation (Alberti et al. 2001; Hahs and McDonnell 2006; McKinney 2008). The specific impacts of urbanisation on species richness vary, depending on variables, such as geographic location and many historical and economic factors that are unique to each city (McKinney 2008).

Urbanisation intensity is correlated with increased disturbance and structural simplification of the remaining vegetation through landscaping practices that remove woody plants, leaf litter and other microhabitats from natural communities (Marzluff and Ewing 2001). Combination of all these factors reduces the area and quality of animal habitat and tends to increase with the intensity of urbanisation (Alberti et al. 2001; Hahs and McDonnell 2006). Studies using spatial gradients in urban areas have shown that the development of these areas can strongly and negatively affect many sensitive butterfly species (Blair and Launer 1997; Blair 1999; Clark et al. 2007). The close relationship between the abundance of host plants and the persistence status of butterflies suggests that the decline of plants may cause the co-extinction of some associated butterflies (Koh et al. 2004) or the host plant’s own rarity (rather than decrease) could be associated with another trait of the butterfly that makes it vulnerable to extirpation (Harrison 1991). Corke (1999), Mulder et al. (2005) and Öckinger et al. (2006) suggest that the butterfly decline in urban areas could be a secondary effect of heavy metal stress presence on local plants, not resulting in a decrease in the number of host-plants, but in a selective pressure of pollutants on the plant vigour, subsequently affecting their associated fauna. Either way, our results corroborate similar studies of declining butterfly populations, suggesting that habitat degradation may be a devastating threat to the persistence of certain sensitive taxa, such as butterflies characteristic of unpolluted and low-pollution sites (Schultz and Dlugosch 1999; Weiss 1999; Wagner and Van Driesche 2010; Bonebrake and Cooper 2014).

A study of butterfly communities in fragments of urban forests in Brazil (Brown and Freitas 2002) similarly found that the most important factors affecting diversity and composition, excluding site size and sampling time, were connectivity, vegetation, flowers and negative human impact, such as pollution (indirect effect of urbanisation). Observations of butterfly diversity provide information on variations in species richness and abundance formed by vegetation throughout the landscape and the interaction between species (Öckinger et al. 2009). Although local determinants of diversity, such as competition and predation, remained undetermined in these studies, to a large extent, landscape characteristics influence butterfly richness and abundance in different geographic areas (Öckinger et al. 2006; Öckinger et al. 2009). Spatial scale differences in butterfly diversity can be attributed to heterogeneity at the landscape level, while timescale differences can be attributed to changes in climatic conditions at both local and regional scales (Mukherjee et al. 2015). In the current context, it can be assumed that butterfly diversity varies in the four sampling sites as a matter of differences in pollutants concentration and composition of vegetation.

In general, the differences in species distribution in the four areas were prominent, although the abundance of the different species was not profound (indirect effect of the high degree of urbanisation), possibly due to the high concentrations of the contaminants, as well as the corresponding abundance of host plants in the affected areas. The observed variations in species richness in areas without apparent pollution provide an impression of differences in food plants abundance and landscape characteristics in the region. Previous studies on the diversity of butterflies in landscapes with high pollution in contrast to the regions of moderate and low pollution show that the richness increased with the availability of green space and the heterogeneity of habitats in terms of the available plant species and dominant microenvironmental conditions (Kuussaari et al. 2007). According to these studies, our observations register a greater diversity in the areas with no apparent pollution and low urbanisation, followed by the areas of low, moderate and high pollution and urbanisation (Blair and Launer 1997; Kitahara and Sei 2001; Hogsden and Hutchinson 2004).

Regardless of variations between different landscapes, observations of butterfly diversity in the study area suggest that conservation management is necessary to ensure the livelihood of the different ecosystem services derived from butterflies. The abundance of butterflies in urban landscapes will promote the pollination and hence the propagation of different plant species that can reduce the decrease in vegetation, consequently diminishing other variables, such as noise and mainly pollution levels (Mukherjee et al. 2015; Selmi et al. 2016; Alfie and Salinas 2017). To understand the ways these insects respond to urbanisation, different authors have suggested three ecological patterns that are related to our results: (1) there are fewer butterfly species in highly urbanised areas (Knapp et al. 2008; Soga et al. 2014); (2) the number of specialised butterflies decreases with increasing urbanisation, a case demonstrated by the number of indicator species in each gradient category (Bergerot et al. 2011; Lizée et al. 2011; Soga and Koike 2012, 2013); and (3) urbanisation can lead to local disappearance of rare and not abundant, specialised butterfly species, as shown in this study (Fattorini 2011; Soga and Koike 2012).

We found that the variables associated with the increase in urbanisation (NO_2_, NO_x_ and PM_2.5_) were negatively correlated with the richness of butterflies, while the measures associated with less developed areas (green space) were positively correlated. These results are consistent with those of Ruszczyk (1986), Ruszczyk and DeAraujo (1992) and Stefanescu et al. (2004), who found lower diversity of species with higher urbanisation. Growing more trees and shrubs that provide nectar for adult butterflies and using a greater variety of larval food plants species in planting schemes, may be more effective in maintaining populations of these insects than simply increasing the amount of plant cover cultivated (Koh and Sodhi 2004).

The richness and distribution of butterfly species fluctuates according to their life cycle, which is linked to seasonal changes. However, compared to butterflies in temperate climates, seasonal variation generally does not have a great impact on tropical butterflies, which are reported as well distributed throughout the year, the case corresponding to the present study, as there is no seasonal differentiation for most of the comparisons (Hamer et al. 2005). In the MMZ, the butterflies showed a highest species richness during the dry season with 88% of the total species observed during the evaluation period. This finding is contrary to other studies, which report higher numbers in the rainy season (Devries et al. 1997; Hamer et al. 2005; Hernández-Mejía et al. 2008; Meléndez-Jaramillo et al. 2019). This can be attributed to human intervention, when irrigation provides higher food resources for butterflies and also attracts a higher number of species than normal during the dry season.

Biodiversity inventories provide crucial reference information for future ecological and conservation studies. The existence of species lists at various stages of the urbanisation process allows documentation of changes in species composition over time. However, few lists of butterfly species have been published in cities, most of which are restricted to few countries, for example, Brasil, Argentina or India (Núñez 2008; Chowdhury and Soren 2011; Silva et al. 2012). Until now, significant efforts have been made in and around cities to conserve endangered butterfly species (Daniels 2009; Ramírez and MacGregor 2016). Butterfly conservation in urban areas is a feasible task, since many species can thrive in these areas. Hopefully, creative urban planning and management, such as habitat design and planting of native, nectar-rich plants, could improve urban habitats for butterflies. However, all actions must be monitored and must build on prior knowledge about the biology and ecology of the target species to be successful (Snep et al. 2006; Kadlec et al. 2008).

## Conclusions

For the first time in Mexico, butterflies were systematically sampled in order to monitor the environmental quality in an urban area. A total of 8,570 specimens belonging to six families, 19 subfamilies, 31 tribes, 138 genera and 209 species of butterflies were collected for the study area. The highest species abundance and richness, as well as alpha diversity, are recorded at the site free from air pollution, that is associated with a less impacted landscape. Both species richness and abundance were significantly different between all sites, except for the comparison between the moderate contamination site and the high contamination site, while diversity decreased significantly with increasing levels of contamination. The overall trend of distribution of butterflies to the levels of air pollution shown in the Monterrey Metropolitan Area is a decrease, this being in agreement with the general disturbance hypothesis.

The seasonality effect was absent on species richness; however, for species abundance, the differences between dry season and rainy season were significant in each site, excepting the moderate contamination site. Regarding diversity, the seasonal effect showed different distribution patterns according to each order. The variables NO_2_, NO_x_, PM_2.5_, relative humidity and vegetation cover, were highly correlated, both with species abundance and richness, so they could be the main reasons for the variation of butterfly communities in this study.

This work is one of the first studies of butterflies in a specific area of northeast Mexico, in which the environmental quality and seasonality in an urban area were analysed. The information presented here provides benchmarks that allow the comparison of the diversity and richness of Papilionoidea species at regional and national levels. This information can be used as an initial step to analyse the possible use of butterflies as an indicator group of the biodiversity in Mexico.

## Appendix 1

**Table:** Taxonomic list of Papilionoidea by season in each pollution category in the Monterrey Metropolitan Area. Legend: S 1 = Site 1 (Pollution free), S 2 = Site 2 (Low pollution), S 3 = Site 3 (Moderate pollution), S 4 = Site 4 (High pollution). The marked species (*) were collected with Van Someren-Rydon traps.

Taxon	Dry season				Rainy season				General (MMZ)
	S 1	S 2	S 3	S 4	S 1	S 2	S 3	S 4	
**Papilionidae** Latreille, 182									
**Papilioninae** Latreille, 182									
**Troidini** Talbot, 1939									
*Parides photinus* (Doubleday, 1844)	3				3				6
*Parides erithalion polyzelus* (C. Felder & R. Felder, 1865)	5								5
*Battus philenor philenor* (Linnaeus, 1771)	8	12	13	8	17	16	10	16	100
*Battus polydamas polydamas* (Linnaeus, 1758)	9	11	11	6	11	17	8	13	86
**Leptocircini** W. F. Kirby, 1896									
*Protographium epidaus epidaus* (Doubleday, 1846)	4								4
*Protographium philolaus philolaus* (Boisduval, 1836)	4	3			5	6			18
**Papilionini** Latreille, 182									
*Papilio polyxenes asterius* (Stoll, 1782)	4	5	10	7	12	7	8	11	64
*Pterourus pilumnus* Boisduval, 1836	12	10			8	9	9	7	55
*Pterourus palamedes leontis* Rothschild & Jordan, 196	10				7				17
*Heraclides cresphontes* Cramer, 1777	12	9	5	10	6	8	9	7	66
*Heraclides thoas autocles* Rothschild & Jordan, 196	8	13							21
*Heraclides astyalus pallas* G. Gray, 1853	7	8	7		12	10	11		55
*Heraclides ornythion* Boisduval, 1836	13	9			5	12	7	8	54
*Heraclides anchisiades idaeus* Fabricius, 1793	8				10				18
**Pieridae** Swainson, 182									
**Coliadinae** Swainson, 1821									
*Kricogonia lyside* (Godart, 1819)	14	14	21	16	23	12	16	26	142
*Nathalis iole iole* Boisduval, 1836	12	17	7	12	20	13	18	9	108
*Eurema daira eugenia* (Wallengren, 186)	13	14			20	11			58
*Eurema boisduvaliana* (C. Felder & R. Felder, 1865)	9	8	16		19				52
*Eurema mexicana mexicana* (Boisduval, 1836)	13	16	11	15		14	15	17	101
*Abaeis nicippe* (Cramer, 1779)	20	12	14	13	14	16	7	9	105
*Pyrisitia proterpia* (Fabricius, 1775)	19	23	9	13	13	10	12	18	117
*Pyrisitia lisa centralis* (Herrich-Schäffer, 1865)	12	10	14	12	7	11	16	16	98
*Pyrisitia nise nelphe* (R. Felder, 1869)	4	14			12	18	18	11	77
*Pyrisitia dina westwoodii* (Boisduval, 1836)	17				21				38
*Colias eurytheme* Boisduval, 1832		9	11	12					32
*Zerene cesonia cesonia* (Stoll, 179)	15	9	16	9	10	19	17	14	109
*Anteos clorinde* (Godart, 1824)	14	9			23	15			61
*Anteos maerula* (Fabricius, 1775)	20	15	16		15	11	9	16	102
*Phoebis sennae marcellina* (Cramer, 1777)	11	19	11	9	20	23	15	12	120
*Phoebis philea philea* (Linnaeus, 1763)	10	6	9		14	13			52
*Phoebis agarithe agarithe* (Boisduval, 1836)	16	11	6	14	7	18	15	13	100
**Pierinae** Swainson, 182									
**Pierini** Swainson, 182									
*Glutophrissa drusilla tenuis* (Lamas, 1981)	8	4	4	5	8	9	6	3	47
*Catasticta nimbice nimbice* (Boisduval, 1836)	6	4			6				16
*Leptophobia aripa elodia* (Boisduval, 1836)	8				11				19
*Pontia protodice* (Boisduval & Le Conte, 183)	7	6			10	13	12	8	56
*Ascia monuste monuste* (Linnaeus, 1764)	4	1	5		7	6	12	13	48
*Ganyra josephina josepha* (Salvin & Godman, 1868)	4	2	5	6					17
**Lycaenidae** Leach, 1815									
**Theclinae** Swainson, 1831									
**Eumaeini** E. Doubleday, 1847									
*Eumaeus childrenae* (G. Gray, 1832)	5				13				18
*Atlides halesus corcorani* Clench, 1942	7								7
*Rekoa zebina* (Hewitson, 1869)	6	6			9				21
*Rekoa marius* (Lucas, 1857)	7	2	5		8	14			36
*Arawacus jada* (Hewitson, 1867)		6	6	6		8	10	5	41
*Ocaria ocrisia* (Hewitson, 1868)			5	5			11	10	31
*Chlorostrymon telea* (Hewitson, 1868)				2					2
*Cyanophrys herodotus* (Fabricius, 1793)	5				10				15
*Cyanophrys miserabilis* (Clench, 1946)	4	10			7	12			33
*Allosmaitia strophius* (Godart, 1824)	8				10				18
*Laothus erybathis* (Hewitson, 1867)	5	4							9
*Electrostrymon guzanta* (Schaus, 192)	5	9	4	8	11	6	13	7	63
*Calycopis isobeon* (Butler & H. Druce, 1872)	6	5	8	8	14	10	10	7	68
*Strymon melinus melinus* Hübner, 1818	3	7	5	8	9	13	10	12	67
*Strymon rufofusca* (Hewitson, 1877)	3	5			10				18
*Strymon albata* (C. Felder & R. Felder, 1865)	3								3
*Strymon bebrycia* (Hewitson, 1868)					13				13
*Strymon yojoa* (Reakirt, 1867)	4	8			5	11			28
*Strymon bazochii bazochii* (Godart, 1824)	2				10				12
*Strymon istapa istapa* (Reakirt, 1867)	9	6	10	1	6	10	9	10	61
*Tmolus echion echiolus* (Draudt, 192)					9				9
*Ministrymon clytie* (W. H. Edwards, 1877)	9	7	8	4	9	14	13	8	72
*Ministrymon azia* (Hewitson, 1873)	3	6	5		15	5			34
*Michaelus hecate* (Godman & Salvin, 1887)					5				5
**Polyommatinae** Swainson, 1827									
*Leptotes cassius cassidula* (Boisduval, 187)	14	11	9	9	4	14	14	11	86
*Leptotes marina* (Reakirt, 1868)	8	7			19	10	13		57
*Brephidium exilis exilis* (Boisduval, 1852)			10	9					19
*Cupido comyntas comyntas* (Godart, 1824)					12	12			24
*Echinargus isola* (Reakirt, 1867)	6	5	12	13	15	15	11	7	84
*Hemiargus ceraunus astenidas* (Lucas, 1857)	10	9	10	3	13	15	9		69
**Riodinidae** Grote, 1895									
**Riodininae** Grote, 1895									
*Calephelis nemesis australis* (W. H. Edwards, 1877)	7	2	9	5	9	2	5	5	44
*Calephelis perditalis perditalis* W. Barnes & McDunnough, 1918	5	5	6	8	11	8	5	12	60
*Calephelis rawsoni* McAlpine, 1939	7	4			7				18
*Caria ino melicerta* Schaus, 189	7	2	8		7	6	7		37
*Lasaia agesilas callaina* Clench, 1972	6	10			7	13			36
*Anteros carausius carausius* Westwood, 1851			4	7					11
*Emesis tenedia* C. Felder & R. Felder, 1861	8	7	5	3	10	5	6	9	53
*Emesis emesia* (Hewitson, 1867)	7	7	6	8	3	10	3	4	48
*Apodemia hypoglauca hypoglauca* (Godman & Salvin, 1878)	2								2
**Nymphalidae** Rafinesque, 1815									
**Libytheinae** Boisduval, 1833									
**Libytheana carinenta larvata* (Strecker, 1878)	11	9	12	14	16	19	16	16	113
**Danainae** Boisduval, 1833									
**Danaini** Boisduval, 1833									
*Danaus plexippus plexippus* (Linnaeus, 1758)	5	5	8	11	7	9	6	10	61
*Danaus gilippus thersippus* (H. Bates, 1863)	7	5	7	7	8	10	6	3	53
*Danaus eresimus montezuma* Talbot, 1943	6	10			11	10			37
**Ithomiini** Godman & Salvin, 1879									
*Pteronymia cotytto* (Guérin-Méneville, 1844)					1				1
**Heliconiinae** Swainson, 1822									
**Heliconiini** Swainson, 1822									
*Agraulis vanillae incarnata* (N. Riley, 1926)	8	5	3	2	5	11	11	10	55
*Dione moneta poeyii* Butler, 1873	5	5							10
*Dryas iulia moderata* (N. Riley, 1926)	7	3	6	7	8	12	11	10	64
*Heliconius charithonia vazquezae* W. Comstock & F. Brown, 195	6	8	11	3	6	7	11	7	59
*Heliconius erato petiverana* (E. Doubleday, 1847)					7				7
**Argynnini** Swainson, 1833									
*Euptoieta claudia* (Cramer, 1775)	6	6		8	11	9	5	9	54
*Euptoieta hegesia meridiania* Stichel, 1938	10	6	10	9	7	8	10	10	70
**Limenitidinae** Behr, 1864									
**Limenitidini** Behr, 1864									
**Limenitis arthemis astyanax* (Fabricius, 1775)	4				3				7
**Adelpha paroeca paroeca* (H. Bates, 1864)					9	8			17
**Adelpha fessonia fessonia* (Hewitson, 1847)	7	9	5	6	9	11	7	16	70
**Adelpha basiloides* (H. Bates, 1865)	9	6			8	3	8		34
**Apaturinae** Boisduval, 184									
**Asterocampa celtis antonia* (W. H. Edwards, 1878)	9	14	7	6	6	7	6	13	68
**Asterocampa leilia* (W. H. Edwards, 1874)	8	11	10	4	7	8	10	15	73
**Asterocampa clyton louisa* D. Stallings & Turner, 1947	4	7	10	9	9	12	8	11	70
**Asterocampa idyja argus* (H. Bates, 1864)	13				6	9			28
**Doxocopa pavon theodora* (Lucas, 1857)	9	11							20
*Doxocopa laure laure* (Drury, 1773)	6	9	9		11	5	10		50
**Biblidinae** Boisduval, 1833									
**Biblidini** Boisduval, 1833									
**Biblis hyperia aganisa* Boisduval, 1836	9	3	6	9	14	12	10	5	68
*Mestra amymone* (Ménétriés, 1857)	10	13	9	7	15	11	5	10	80
**Catonephelini** Orfila, 1952									
**Eunica tatila tatila* (Herrich-Schäffer, 1855)	10	5	10		9	14			48
**Eunica monima* (Stoll, 1782)	9	10			10				29
**Myscelia ethusa ethusa* (Doyère, 184)	9	9	8	8	7	8	4	8	61
**Ageroniini** E. Doubleday, 1847									
**Hamadryas februa ferentina* (Godart, 1824)	9	6	5	5	10	13	11	10	69
**Hamadryas glauconome glauconome* (H. Bates, 1864)	4	4	11		5	5			29
**Epiphelini** Jenkins, 1987									
**Epiphile adrasta adrasta* Hewitson, 1861	2	3			7				12
**Temenis laothoe* (Cramer, 1777)					4				4
**Eubagini** Burmeister, 1878									
*Dynamine postverta mexicana* d’Almeida, 1952	5	4	2	6	3	5	3	5	33
**Cyrestinae** Guenée, 1865									
**Cyrestini** Guenée, 1865									
*Marpesia petreus* (Cramer, 1776)	3	3			1				7
**Nymphalinae** Rafinesque, 1815									
**Nymphalini** Rafinesque, 1815									
*Vanessa virginiensis* (Drury, 1773)	6	5			6				17
*Vanessa cardui* (Linnaeus, 1758)	8	10	4		10	8	8	6	54
**Vanessa atalanta rubria* (Fruhstorfer, 199)	3	3	2	6	3	6	4	4	31
**Polygonia interrogationis* (Fabricius, 1798)	1	5							6
**Victorinini** Scudder, 1893									
*Anartia jatrophae luteipicta* (Fruhstorfer, 197)	3	3	6	4	9	7	6	10	48
*Anartia fatima fatima* (Fabricius, 1793)	4	6	6	5	6	11	9	9	56
*Siproeta stelenes biplagiata* (Fruhstorfer, 197)	7	4	7	5	4	11	10	13	61
**Junoniini** Reuter, 1896									
*Junonia coenia coenia* Hübner, 1822		4	1	1		4	3	4	17
**Melitaeini** Newman, 187									
*Chlosyne janais janais* (Drury, 1782)	10	9	8	8	5	13	16	15	84
*Chlosyne definita definita* (E. Aaron, 1885)	3	9	7	7					26
*Chlosyne endeis pardelina* Scott, 1986	11	8	7		9	8	7		50
*Chlosyne rosita browni* Bauer, 1961	8	13	7	9	10	11	4	11	73
*Chlosyne theona bollii* (W. H. Edwards, 1877)	9	12	7	13	5	14	17		77
*Chlosyne lacinia adjutrix* Scudder, 1875	7	8	5	10	12	15	11	13	81
*Microtia elva elva* H. Bates, 1864	12	4	7	11	17	13	9	10	83
*Dymasia dymas dymas* (W. H. Edwards, 1877)	8				10				18
*Texola elada ulrica* (W. H. Edwards, 1877)	5	9	8	10					32
*Anthanassa texana texana* (W. H. Edwards, 1863)	11	7	12	7	11	14	12	17	91
*Anthanassa ardys* (Hewitson, 1864)	12				12				24
*Anthanassa ptolyca* (H. Bates, 1864)	10	10	8						28
*Anthanassa argentea* (Godman & Salvin, 1882)	6	7	12	7	15	12	9	18	86
*Anthanassa tulcis* (H. Bates, 1864)					16	13			29
*Phyciodes graphica* (R. Felder, 1869)	6	8	8	10					32
*Phyciodes phaon phaon* (W. H. Edwards, 1864)	11	13	6						30
*Phyciodes tharos tharos* (Drury, 1773)	6	10	8	9	5	12	12	14	76
**Charaxinae** Guenée, 1865									
**Anaeini** Reuter, 1896									
**Anaea aidea* (Guérin-Méneville, 1844)	15	11	12	12	12	20	22	18	122
**Fountainea glycerium glycerium* (E. Doubleday, 1849)	2	5	3		9	6			25
**Memphis pithyusa pithyusa* (R. Felder, 1869)	5				4				9
**Satyrinae** Boisduval, 1833									
**Satyrini** Boisduval, 1833									
**Cyllopsis dospassosi* L. Miller, 1974	3				4	7			14
**Cyllopsis gemma freemani* (D. Stallings & Turner, 1947)	7	8	7	8	20	14	17	13	94
**Megisto rubricata rubricata* (W. H. Edwards, 1871)	3								3
*Hermeuptychia hermes* (Fabricius, 1775)	8	9	8	8	11	13	5	8	70
**Hesperiidae** Latreille, 189									
**Eudaminae** Mabille, 1877									
*Phocides polybius lilea* (Reakirt, 1867)	1								1
*Phocides urania urania* (Westwood, 1852)	4	2			1	5			12
*Polygonus leo arizonensis* (Skinner, 1911)					2				2
*Chioides albofasciatus* (Hewitson, 1867)	6	5	5	8	5	6	8	11	54
*Chioides zilpa* (Butler, 1872)	6	5	7	8	5	7	15	9	62
*Aguna asander asander* (Hewitson, 1867)		3	1	4		4	2	4	18
*Aguna metophis* (Latreille, 1824)	2	5	3	3					13
*Typhedanus undulatus* (Hewitson, 1867)					1	1	3		5
*Urbanus proteus proteus* (Linnaeus, 1758)	7	4	7		6	11	11	10	56
*Urbanus dorantes dorantes* (Stoll, 179)	7	6	7		10	7	7		44
*Urbanus procne* (Plötz, 1881)	8	8	9	5	11	14	8	4	67
*Astraptes fulgerator azul* (Reakirt, 1867)	3	6	7	6	11	7	15	9	64
*Astraptes alector hopfferi* (Plötz, 1881)					7	10			17
*Astraptes anaphus annetta* Evans, 1952					2				2
*Autochton cellus* (Boisduval & Le Conte, 1837)	6				7				13
*Autochton cincta* (Plötz, 1882)	1	4	9		6	5	5		30
*Autochton neis* (Geyer, 1832)	7	4	6	7					24
*Achalarus toxeus* (Plötz, 1882)	8	5	9	4	5	5	3	8	47
*Thorybes pylades albosuffusa* H. Freeman, 1943					6				6
*Cabares potrillo potrillo* (Lucas, 1857)	8	7	9	7	3	8	5	7	54
*Spathilepia clonius* (Cramer, 1775)		2	4	5					11
*Cogia hippalus hiska* Evans, 1953	3	8	7	7					25
**Pyrginae** Burmeister, 1878									
**Carcharodini** Verity, 194									
*Arteurotia tractipennis tractipennis* Butler & H. Druce, 1872	7	4			5	7			23
*Polyctor enops* (Godman & Salvin, 1894)	3				7				10
*Noctuana lactifera bipuncta* (Plötz, 1884)					7	5			12
*Bolla brennus brennus* (Godman & Salvin, 1896)	4	6			6	4	5		25
*Staphylus mazans* (Reakirt, 1867)	8	6	4	8	10	6	11	14	67
*Staphylus azteca* (Scudder, 1872)	7				6				13
*Pholisora catullus* (Fabricius, 1793)	3	7	6	4	7	8	8	11	54
**Erynnini** Brues & F. Carpenter, 1932									
*Gorgythion begga pyralina* (Möschler, 1877)	9	4	5	3	7	9	4	7	48
*Sostrata nordica* Evans, 1953	7								7
*Grais stigmaticus stigmaticus* (Mabille, 1883)	8	9	6		8	3			34
*Timochares ruptifasciata* (Plötz, 1884)					10				10
*Chiomara georgina georgina* (Reakirt, 1868)	5	6	5	9	7	14	9	13	68
*Gesta invisus* (Butler & H. Druce, 1872)	4	7	3	6	2	6	8	5	41
*Erynnis funeralis* (Scudder & Burgess, 187)	1	3	6	8					18
**Achlyodidini** Burmeister, 1878									
*Eantis tamenund* (W. H. Edwards, 1871)	5	6	10	5	12	7	11	8	64
*Zera hyacinthinius hyacinthinus* (Mabille, 1877)	5	7	6	6	4				28
**Pyrgini** Burmeister, 1878									
*Carrhenes canescens canescens* (R. Felder, 1869)	3								3
*Systasea pulverulenta* (R. Felder, 1869)	7	8	9	6	6	7	6	4	53
*Celotes nessus* (W. H. Edwards, 1877)			8	6			4	6	24
*Pyrgus albescens* Plötz, 1884	8	10	7	3	12	8	13	16	77
*Pyrgus oileus* (Linnaeus, 1767)	6	11	6	5	22	7	14	19	90
*Pyrgus philetas* W. H. Edwards, 1881	1	1	1	3					6
*Heliopyrgus sublinea* (Schaus, 192)	6	6	7		8	5			32
*Heliopetes laviana laviana* (Hewitson, 1868)	9	5	5	5	8	4	9	8	53
*Heliopetes macaira macaira* (Reakirt, 1867)					9				9
**Hesperiinae** Latreille, 189									
**Thymelicini** Tutt, 195									
*Ancyloxypha arene* (W. H. Edwards, 1871)	7	7	3	7	12	4	2	9	51
*Copaeodes aurantiaca* (Hewitson, 1868)	4	3	6	4	7	11	3	10	48
*Copaeodes minima* (W. H. Edwards, 187)	2	5	5	6	12	12	10	10	62
**Calpodini** A. Clark, 1948									
*Panoquina lucas* (Fabricius, 1793)					2	5			7
**Anthoptini** A. Warren, 29									
*Synapte pecta* Evans, 1955					1				1
**Moncini** A. Warren, 28									
*Remella rita* (Evans, 1955)					2				2
*Amblyscirtes tolteca tolteca* Scudder, 1872	2								2
*Cymaenes trebius* (Mabille, 1891)					5				5
*Lerodea eufala eufala* (W. H. Edwards, 1869)	4	3	8		4	6			25
*Lerema accius* (J. E. Smith, 1797)	5	5	3	6	6	10	9	7	51
*Lerema liris* Evans, 1955	5	7	4	5	7	3	6	6	43
*Vettius fantasos* (Cramer, 178)					2	4			6
**Hesperiini** Latreille, 189									
*Hylephila phyleus phyleus* (Drury, 1773)	3	3	3	2	3	5	8	7	34
*Polites vibex praeceps* (Scudder, 1872)	4	5	3	4	7	7	2	5	37
*Wallengrenia otho otho* (J. E. Smith, 1797)					4				4
*Atalopedes campestris huron* (W. H. Edwards, 1863)	1	2	4	3	5	4	4	4	27
*Poanes melane vitellina* (Herrich-Schäffer, 1869)	4	4							8
*Anatrytone mazai* (H. Freeman, 1969)					3				3
*Quasimellana eulogius* (Plötz, 1882)	4	2				3	4	3	16
*Quinta cannae* (Herrich-Schäffer, 1869)					6	2			8
*Nyctelius nyctelius nyctelius* (Latreille, 1824)	5	3	5	6	6	9	7	9	50
